# Up‐regulation of paired‐related homeobox 2 promotes cardiac fibrosis in mice following myocardial infarction by targeting of Wnt5a

**DOI:** 10.1111/jcmm.14914

**Published:** 2019-12-27

**Authors:** Wen‐Wu Bai, Zhen‐Yu Tang, Ti‐Chao Shan, Xue‐Jiao Jing, Peng Li, Wei‐Dong Qin, Ping Song, Bo Wang, Jian Xu, Zhan Liu, Hai‐Ya Yu, Zhi‐Min Ma, Shuang‐Xi Wang, Chao Liu, Tao Guo

**Affiliations:** ^1^ The Key Laboratory of Cardiovascular Remodeling and Function Research Chinese Ministry of Education Chinese National Health Commission and Chinese Academy of Medical Sciences The State and Shandong Province Joint Key Laboratory of Translational Cardiovascular Medicine Qilu Hospital of Shandong University Jinan China; ^2^ Department of Traditional Chinese Medicine Qilu Hospital of Shandong University Jinan China; ^3^ Department of Emergency Qilu Hospital of Shandong University Jinan China; ^4^ Department of Critical Care Medicine Qilu Hospital of Shandong University Jinan China; ^5^ Department of Geriatric Medicine Qilu Hospital of Shandong University, Key Laboratory of Cardiovascular Proteomics of Shandong Province Qilu Hospital of Shandong University Jinan China; ^6^ Department of Pharmacology College of Pharmacy Xinxiang Medical University Xinxiang China; ^7^ Department of Gastroenterology and Clinical Nutrition The First Affiliated Hospital of Hunan Normal University Changsha China; ^8^ Department of Neurology The People’s Hospital of Xishui County Huangang China; ^9^ Department of Endocrinology The Affiliated Suzhou Science & Technology Town Hospital of Nanjing Medical University Suzhou China; ^10^ Hubei Key Laboratory of Cardiovascular, Cerebrovascular, and Metabolic Disorders Hubei University of Science and Technology Xianning China

**Keywords:** cardiac fibrosis, cell differentiation, myocardial infarction, paired‐related homeobox 2, Wnt5a

## Abstract

Cardiac fibrosis is a key factor to determine the prognosis in patient with myocardial infarction (MI). The aim of this study is to investigate whether the transcriptional factor paired‐related homeobox 2 (Prrx2) regulates Wnt5a gene expression and the role in myocardial fibrosis following MI. The MI surgery was performed by ligation of left anterior descending coronary artery. Cardiac remodelling was assessed by measuring interstitial fibrosis performed with Masson staining. Cell differentiation was examined by analysis the expression of alpha‐smooth muscle actin (α‐SMA). Both Prrx2 and Wnt5a gene expressions were up‐regulated in mice following MI, accompanied with increased mRNA and protein levels of α‐SMA, collagen I and collagen III, compared to mice with sham surgery. Adenovirus‐mediated gene knock down of Prrx2 increased survival rate, alleviated cardiac fibrosis, decreased infarction sizes and improved cardiac functions in mice with MI. Importantly, inhibition of Prrx2 suppressed ischaemia‐induced Wnt5a gene expression and Wnt5a signalling. In cultured cardiac fibroblasts, TGF‐β increased gene expressions of Prrx2 and Wnt5a, and induced cell differentiations, which were abolished by gene silence of either Prrx2 or Wnt5a. Further, overexpression of Prrx2 or Wnt5a mirrored the effects of TGF‐β on cell differentiations of cardiac fibroblasts. Gene silence of Wnt5a also ablated cell differentiations induced by Prrx2 overexpression in cardiac fibroblasts. Mechanically, Prrx2 was able to bind with Wnt5a gene promoter to up‐regulate Wnt5a gene expression. In conclusions, targeting Prrx2‐Wnt5a signalling should be considered to improve cardiac remodelling in patients with ischaemic heart diseases.

## 1 INTRODUCTION

Myocardial infarction (MI) is a leading cause of sudden death.^1^ In response to MI, myocardial fibrosis is caused by aberrantly activated cardiac fibroblasts, excessive accumulation of collagen fibres in the extracellular matrix of the heart muscle, increased collagen concentration or imbalanced collagen composition.^2^, ^3^, ^4^ The extracellular matrix is mainly composed of type I collagen and type III collagen, accounting for more than 90% of the total amount of myocardial interstitial collagen.^5^ Maintenance of proper ratio of myocardial interstitial collagen is important for the integrity of cardiac function.^6^ Molecular mechanisms that underlie cardiac fibrotic disorders are still mostly unclear, and no specific therapies exist for treatment of myocardial fibrosis.

The importance of Wnt signalling in developmental processes like cell proliferation, differentiation and migration has been recognized for decades. Wnt signal is relatively silent in adult heart tissue,^7^ but re‐activated after a variety of cardiac injuries ranging from acute ischaemic insult to chronic pressure overloading.^8^ Abnormal Wnt pathway activation contributes to transforming growth factor‐beta (TGF‐β)‐induced fibrotic remodelling in numerous adult fibrotic diseases including cardiac fibrosis after MI.^9^ Wnt pathway has revealed new points of intervention that may lead to novel drug targets for small molecular weight compounds.^10^, ^11^ However, how Wnt signalling is dysregulated in post‐ischaemic heart needs further investigations.

Paired‐related homeobox genes, including Prrx1 and Prrx2, are members of a subfamily of homeobox genes, which promote transcriptional activation by functional studies.^12^ Mice that lack both Prrx1 and Prrx2 have profound defects in mesenchymal cell differentiation in the craniofacial region.^13^ In vascular system, Prrx1 and Prrx2 are involved matrix modulation^14^ and inhibits adipogenesis through regulated expression of TGF‐β ligands, thereby activating TGF‐β signalling.^15^ Prrx2 has been reported to be induced by TGF‐β and to increase the invasiveness of multiple cancer types through epithelial‐mesenchymal transition.^16^ However, the role of Prrx2 in ischaemia‐induced cardiac remodelling is currently unknown.

As reported, both Prrx2 and Wnt5a were able to be up‐regulated by TGF‐β in myofibroblast and cancer cell.^16^, ^17^ Therefore, we have been suggested that Wnt5a gene expression is directly up‐regulated by Prrx2, which mediates cardiac fibrosis and delays the recovery of cardiac functions after MI. In this study, our findings indicate that Prrx2 was activated in heart tissue after ischaemia, thereby up‐regulating Wnt5a signalling through gene transcription. Moreover, TGF‐β induced cell differentiations of cardiac fibrosis via Prrx2‐dependent Wnt5a signalling activation. Clinically, inhibition of Prrx2 or Wnt5a is an effective approach to improve cardiac remodelling in patients with ischaemic heart diseases.

## 2 MATERIALS AND METHODS

An expanded Methods section is included in the Supporting Information.

### 2.1 Myocardial infarction (MI)

The surgery of MI was operated by ligation of left anterior descending coronary artery (LADCA) as described previously.^18^, ^19^

### 2.2 Echocardiography

Echocardiography with standard parasternal and apical views was conducted in the left lateral recumbent position as we described previously.^20^ Systolic or diastolic left ventricular internal diameter (LVDs or LVDd) were measured. Ejection fraction (EF) and fractional shortening (FS) were calculated.

### 2.3 Statistical analysis

All quantitative results are expressed as mean ± SD. The normal distribution of data was tested by the Kolmogorov‐Smirnov test before statistical comparisons, and the normality/equal variance was tested to determine whether ANOVA was appropriate. Multiple comparisons were analysed with a one‐way ANOVA followed by Tukey post hoc tests or Bonferroni post hoc analyses. Comparisons between two groups were analysed by unpaired Student's *t* test between two groups. Chi‐square test was applied to comparisons of survival rates. Statistical analyses were conducted using GraphPad Prism 6.0 or IBM SPSS statistics 20.0. A two‐sided *P*‐value < .05 was considered significant.

## 3 RESULTS

### 3.1 Both Prrx2 and Wnt5a gene expressions are up‐regulated by ischaemia in *Apoe^−/−^* mice

**Figure 1:**
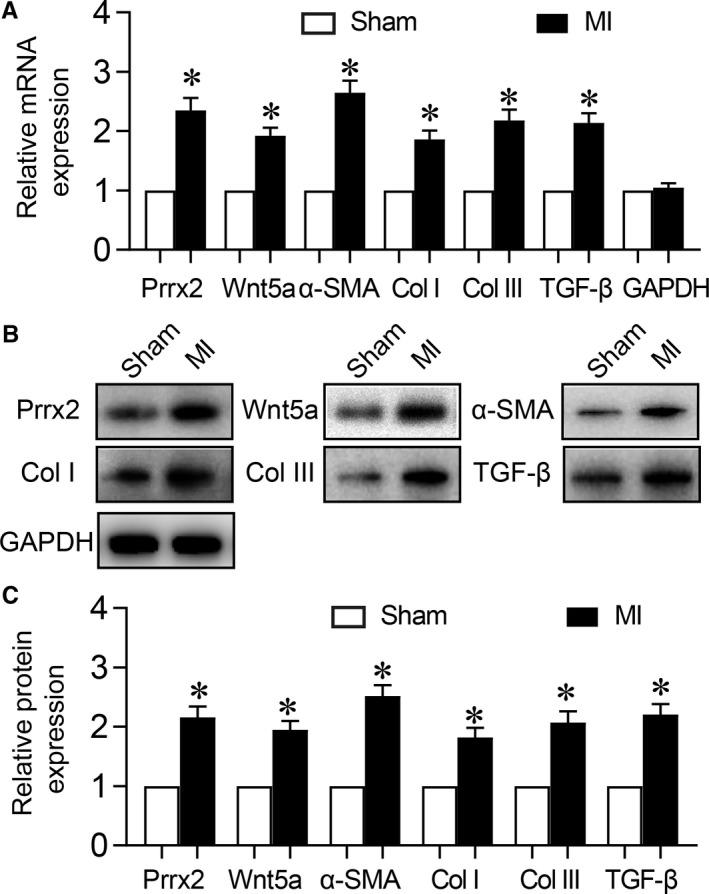
Ischaemia up‐regulates Prrx2 and Wnt5a gene expressions in mice following MI. Male *Apoe^−/−^* mice were subjected to perform MI surgery and hearts were isolated from mice at the 30th post‐operative days to detect (A) gene expressions of Prrx2, Wnt5a, α‐SMA, GAPDH, collagen I (Col I) and collagen III (Col III) by real‐time PCR. B and C, Protein levels of Prrx2, Wnt5a, α‐SMA, GAPDH, collagen I and collagen III in heart homogenates were determined by Western blotting in B and quantitative analysis was performed in C. N is 10‐15 in each group. *P < .05 vs Sham

To investigate the roles of Prrx2 and Wnt5a in cardiac fibrosis, we firstly established the MI model in *Apoe^−/−^* mice by performing the surgery of LADCA ligation. Both mRNA and protein levels of TGF‐β, Prrx2 and Wnt5a were increased in ischaemic hearts at the 30th post‐operative day in the MI model, compared to sham (Figure 1A‐C). While, the plasms levels of total cholesterol (TC), triglyceride (TG), low‐density lipoprotein (LDL) and high‐density lipoprotein (HDL) were comparable between mice with MI surgery and sham (Table S2).

Myocardial fibrosis, defined by the diffuse and disproportionate accumulation of collagen type I and III fibres in the interstitium, represents a final common lesion following a variety of myocardial injuries.^21^ Thus, we detected the contents of collagen I and III, such as mRNA and protein in post‐ischaemic hearts. As expected, both collagen I and III were increased in hearts after ischaemia (Figure 1A‐C). Importantly, we also observed that the levels of α‐SMA mRNA and protein were increased in ischaemic hearts after 30 post‐operative days, compared to heart without ischaemia (Figure 1A‐C).

### 3.2 Adenovirus‐mediated gene knockdown of Prrx2 improves cardiac functions in mice following MI

**Figure 2:**
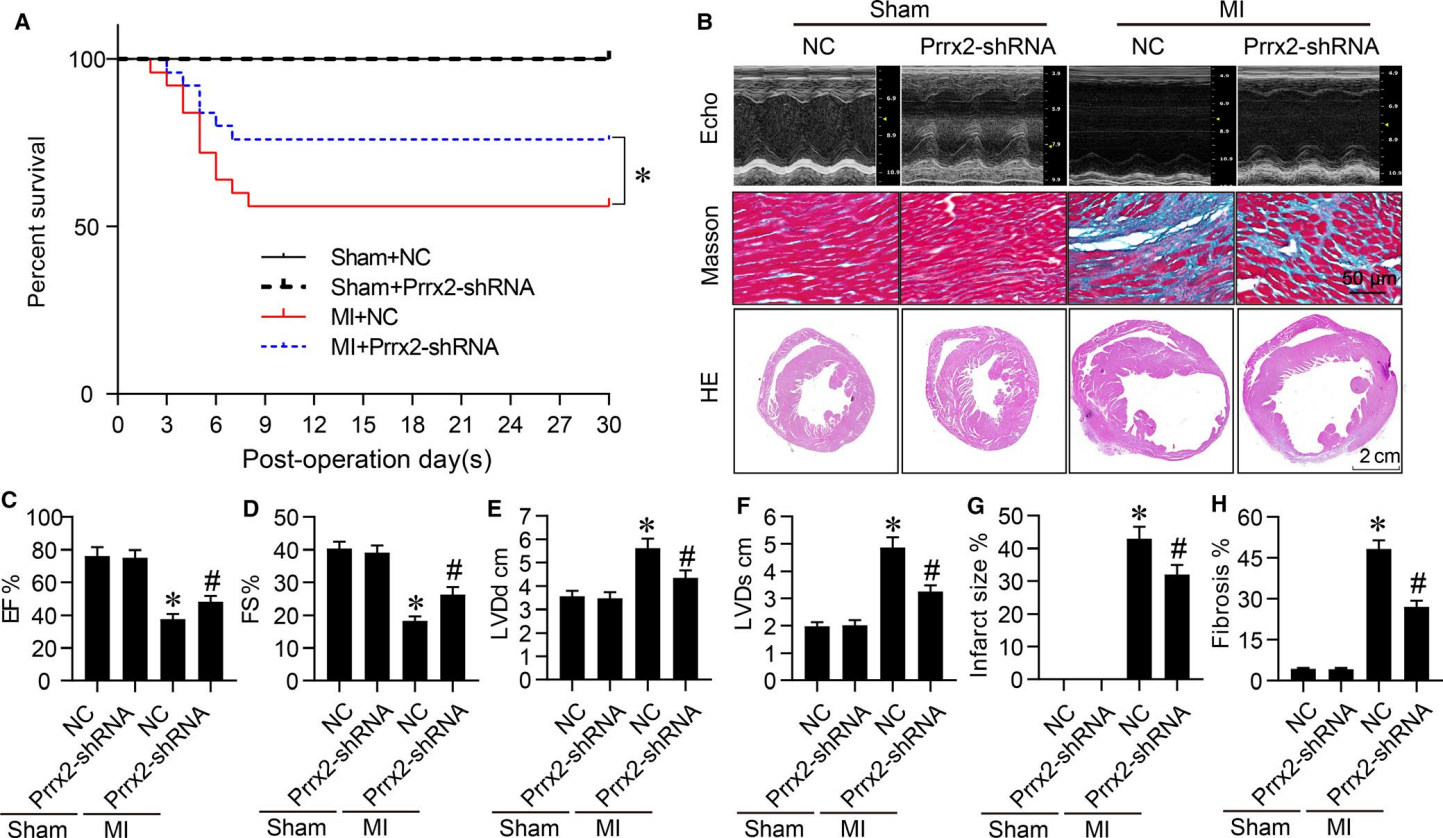
Gene knockdown of Prrx2 inhibits cardiac fibrosis and improves heart functions in mice after MI. Male *Apoe^−/−^* mice were injected with adenovirus expressing negative control (NC) shRNA or Prrx2 shRNA for 3 d prior to MI surgery. At the 30th post‐operative day, mice were subjected to assess cardiac functions by echocardiography before sacrificed and hearts were isolated to measure infraction sizes by HE staining and collagens by Masson staining. A, The survival curve of mice within 30 d after MI surgery. B, Representative images of HE staining, Masson staining and cardiac functions in hearts were shown. C‐F, Cardiac functions were quantified by calculating EF in C, FS in D, measuring LVDd in E and LVDs in F. G, Quantitative analysis of infarct size was performed. H, Quantitative analysis of cardiac fibrosis was conducted. N is 10‐15 in each group. *P < .05 vs NC plus Sham. ^#^ *P* < .05 vs NC plus MI

We next investigated whether Prrx2 up‐regulation is beneficial or detrimental in *Apoe^−/−^* mice following MI. As depicted in Figure 2A, the survival rates in mice undergoing MI surgery were decreased throughout 0‐30 post‐operative days, compared with mice undergoing sham surgery. While, the decreased survival rates in mice with MI surgery were balanced by infecting mice with adenovirus expressing Prrx2 shRNA. Globally, the levels of total cholesterol, triglyceride, low‐density lipoprotein and high‐density lipoprotein in bloods collected from mice were identical among the four groups (Table S3).

We also examined heart functions at the 30th post‐operative day by echocardiography (Figure 2B). The ligation of LADCA dramatically decreased EF (Figure 2C) and FS (Figure 2D), and increased LVDd (Figure 2E) and LVDs (Figure 2F) in mice with MI, compared with mice with sham surgery. Much more importantly, the impaired heart functions in mice with MI surgery were rescued by infecting mice with adenovirus expressing Prrx2 shRNA, compared to negative control shRNA.

### 3.3 Prrx2 deficiency alleviates cardiac fibrosis in mice after MI

Cardiac fibrosis is a key factor to affect the recovery of heart functions in patients with ischaemia heart diseases.^22^ As shown in Figure 2B,G, the infarction sizes in mice injected with adenovirus expressing Prrx2 shRNA were smaller than mice injected with adenovirus expressing negative control shRNA at the 30th day after MI surgery. Prrx2 shRNA reduced the serious degree of interstitial fibrosis, as determined by Masson staining at the 30th post‐operative days (Figure 2B,H), suggesting that Prrx2 up‐regulation is vital to mediate ischaemia‐induced cardiac fibrosis in heart.

### 3.4 Down‐regulation of Prrx2 inactivates Wnt5a signalling in ischaemic heart in mice

**Figure 3:**
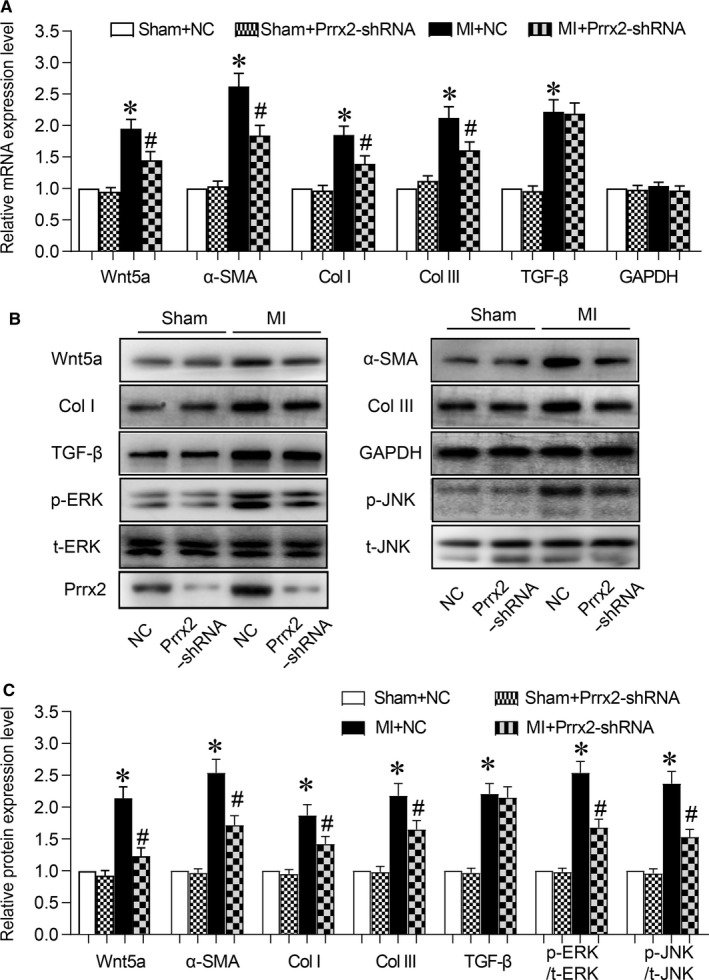
Adenovirus‐mediated Prrx2 shRNA expression down‐regulates Wnt5a signalling in ischaemic hearts in mice. Male *Apoe^−/−^* mice were injected with adenovirus expressing negative control (NC) shRNA or Prrx2 shRNA for 3 d prior to MI surgery. At the 30th post‐operative day, mice were killed under anaesthesia and hearts were isolated to measure (A) gene expressions of Wnt5a, α‐SMA, GAPDH, collagen I (Col I) and collagen III (Col III) by real‐time PCR. B and C, Protein levels of Wnt5a, α‐SMA, TGF‐β, p‐ERK, p‐JNK, collagen I and collagen III in isolated hearts from mice were determined by Western blotting in B and quantitative analysis was performed in C. N is 10‐15 in each group. *P < .05 vs NC plus Sham. ^#^ *P* < .05 vs NC plus MI

Next, the effects of Prrx2 shRNA on Wnt signalling in *Apoe^−/−^* mice with MI were investigated by us. As illustrated in Figure 3A‐C, MI induced both mRNA and protein expressions of TGF‐β, Wnt5a, α‐SMA, collagen I and collagen III in mice infected with adenovirus expressing negative control shRNA, compared to mice with sham surgery. However, the effects of MI on the levels of mRNA and protein expressions of these Wnt5a downstream factors were abolished by Prrx2 gene knockdown in mice infected with adenovirus expressing Prrx2 shRNA. Further, the levels of p‐ERK and p‐JNK were up‐regulated by MI but reduced by down‐regulation of Prrx2 (Figure 3B,C).

### 3.5 TGF‐β induces cell differentiation and activates Prrx2‐Wnt5a signalling in cardiac fibroblasts

**Figure 4:**
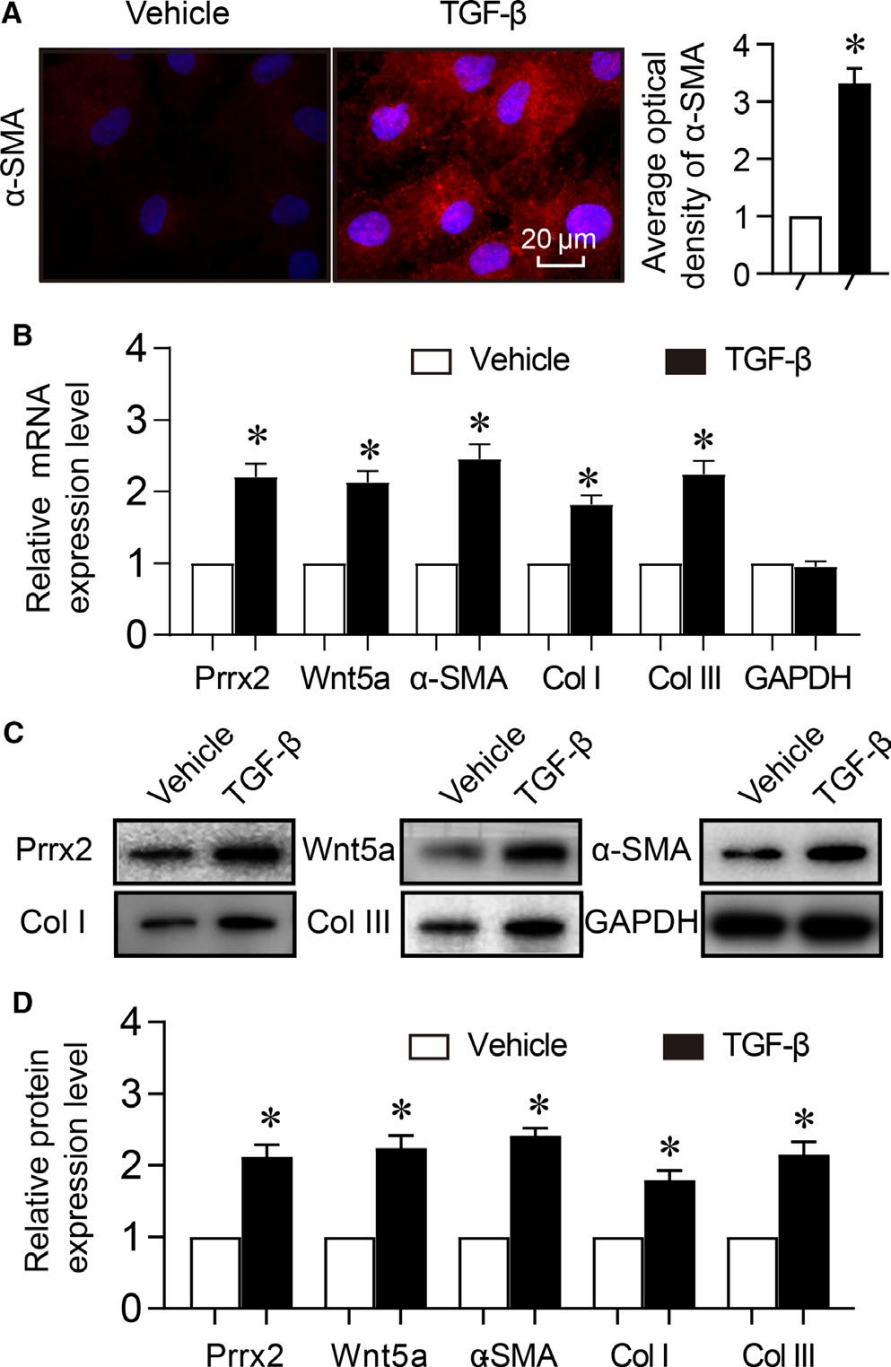
TGF‐β induces cell differentiation and up‐regulates Prrx2 and Wnt5a in cardiac fibroblasts. Cultured cardiac fibroblasts were treated with TGF‐β (10 ng/mL) for 24 h. A, Cell differentiation was determined by immunofluorescence analysis of α‐SMA and quantitative analysis was shown. B, Gene expressions of Prrx2, Wnt5a, α‐SMA, GAPDH, collagen I (Col I) and collagen III (Col III) in cells were measured by real‐time PCR. C and D, Total cell lysates of cardiac fibroblasts were subjected to perform Western blotting analysis to detect protein levels of Prrx2, Wnt5a, α‐SMA, Col I and Col III in C and quantitative analysis was performed in D. N is 5 in each group. *P < .05 vs Vehicle

It has been reported that TGF‐β induces cell differentiation of cardiac fibroblasts to play a crucial role in cardiac fibrogenesis.^23^, ^24^ Then, we examined whether TGF‐β activates Prrx2 and Wnt5a in cardiac fibroblasts by treating cells with TGF‐β. As shown in Figure 4A‐D, TGF‐β induced cell differentiation of cardiac fibroblasts, accompanied with increased Prrx2 gene expression and Wnt5a signalling including Wnt5a, collagen I and collagen III in cultured cardiac fibroblasts, compared to vehicle‐treated cells.

### 3.6 Cell differentiation induced by TGF‐β is Prrx2 dependent in cardiac fibroblasts

**Figure 5:**
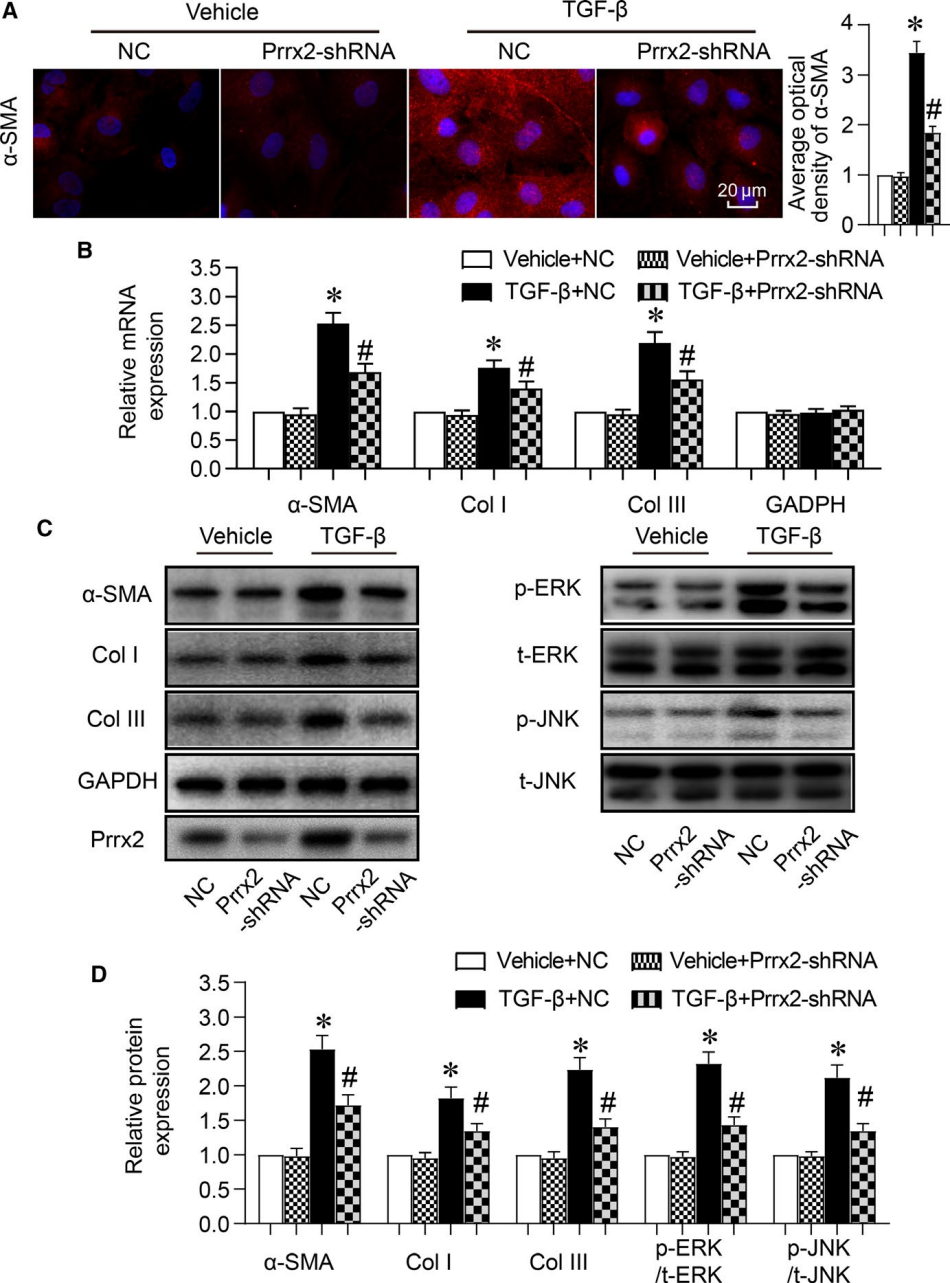
Cell differentiation induced by TGF‐β is abolished by gene silence of Prrx2 in cardiac fibroblasts. Cultured cardiac fibroblasts were infected with adenovirus expressing negative control (NC) shRNA or Prrx2 shRNA for 48 h and then treated with TGF‐β (10 ng/mL) for 24 h. A, Cell differentiation was determined by immunofluorescence analysis of α‐SMA and quantitative analysis was shown. B, Gene expressions of α‐SMA, GAPDH, collagen I (Col I) and collagen III (Col III) in cells were measured by real‐time PCR. C and D, Total cell lysates of cardiac fibroblasts were subjected to perform Western blotting analysis to detect protein levels of α‐SMA, p‐ERK, p‐JNK, Col I, and Col III in C and quantitative analysis was performed in D. N is 5 in each group. *P < .05 vs NC plus Vehicle. ^#^ *P* < .05 vs NC plus TGF‐β

We next determined whether Prrx2 up‐regulated by TGF‐β contributes to cell differentiation of cardiac fibroblasts by infecting cells with adenovirus expressing Prrx2 shRNA to silence the gene expression. As presented in Figure5A‐D, in cells infected with adenovirus expressing negative control shRNA, TGF‐β increased both mRNA and protein expressions of α‐SMA, collagen I and collagen III, compared to cells treated with vehicle. While, these alterations induced by TGF‐β in cardiac fibroblasts were abrogated if Prrx2 gene expression was inhibited through adenovirus‐mediated shRNA. Besides, the levels of p‐ERK and p‐JNK were up‐regulated by TGF‐β but reduced by Prrx2 shRNA (Figure 5C,D).

Cell differentiation in cardiac fibroblasts regulated by Prrx2 was further confirmed by infected cardiac fibroblasts with adenovirus expressing Prrx2 cDNA for 48 hours. As expected, Prrx2 overexpression mimicked the effects of TGF‐β by increasing the mRNA and protein expressions of α‐SMA, collagen I and collagen III in cells, compared to cells infected with adenovirus vector alone (Figure S1A‐D). In sum, it supports the notion that cell differentiation of cardiac fibroblast induced by TGF‐β is Prrx2 dependent.

### 3.7 Wnt5a signalling is involved in cell differentiation in cardiac fibroblasts

The role of Wnt5a signalling in TGF‐β‐induced cell differentiation of cardiac fibroblasts was also investigated by infecting cells with adenovirus expressing Wnt5a shRNA or Wnt5a cDNA. As presented in Figure S2A‐D, TGF‐β increased both mRNA and protein expressions of α‐SMA, collagen I and collagen III in cells infected with adenovirus expressing negative control shRNA, but not in cells infected with adenovirus expressing Wnt5a shRNA. Moreover, Wnt5a overexpression replicated the effects of TGF‐β or Prrx2 overexpression, as observed by increased expressions of these genes in cardiac fibroblasts, compared to cells without overexpressed Wnt5a (Figure S3A‐D).

As reported, Wnt5a has an autocrine or paracrine effect.^25^ We determined if exogenous wnt5a produces the same induction of gene expression. As shown in Figure S3E‐H, similar to Wnt5a overexpression, cardiac fibroblasts treated with Wnt5a recombinant protein increased both mRNA and protein expressions of α‐SMA, collagen I and collagen III, compared to cells treated with vehicle, indicating that Wnt5a may play the role in heart through an autocrine or paracrine effect.

### 3.8 Prrx2 functions as a transcriptional factor to regulate Wnt5a gene expression

**Figure 6:**
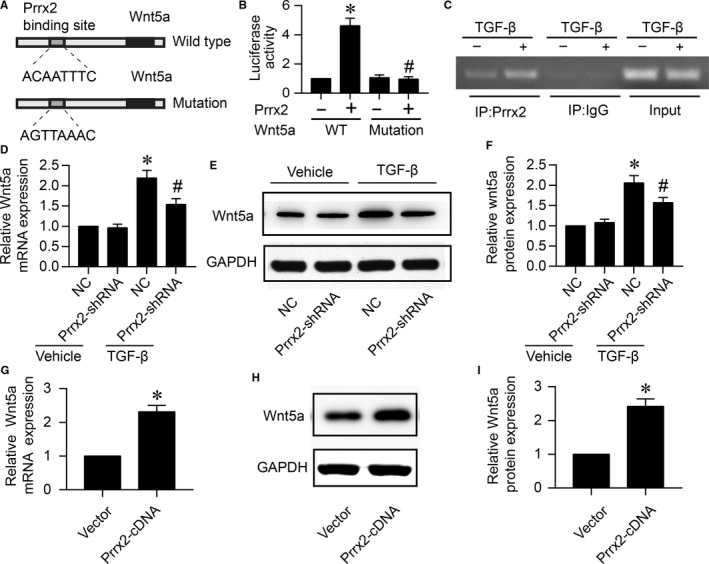
Prrx2 functions as a transcriptional factor of Wnt5a which is up‐regulated by TGF‐β. A, The prediction of binding site for Prrx2 in Wnt5a promoter. B, HEK293A cells were cotransfected with pGL3‐promotor vector constructs expressing WT‐Wnt5a‐promoter containing ACAATTTC or MT‐Wnt5a‐promoter including AGTTAAAC plus Prrx2 cDNA plasmid. Cells were subjected to detect the relative luciferase activity. N is 5 per group. *P < .05 vs WT‐Wnt5a‐promoter. ^#^ *P* < .05 vs WT‐Wnt5a‐promoter plus Prrx2. C, Cultured cardiac fibroblasts were treated with TGF‐β (10 ng/mL) for 24 h. Cells were subjected to detect the binding of Prrx2 to Wnt5a gene promoter by using ChIP method. For ChIP experiment, complex of chromatin/protein was pulled down by Prrx2 primary antibody. The promoter of Wnt5a was amplified by PCR. Positive control is the 10% of the total chromatin in the absence of immunoprecipitation. Negative control is the chromatin immunoprecipitated with IgG and amplified with Wnt5a promoter primers. The PCR production is 200 bp. D‐F, Cultured cardiac fibroblasts were infected with adenovirus expressing negative control (NC) shRNA or Prrx2 shRNA for 48 h and then treated with TGF‐β (10 ng/mL) for 24 h. Total cell lysates were subjected to detect gene expression of Wnt5a by real‐time PCR in D and protein level of Wnt5a by Western blotting in E. Quantitative analysis of Wnt5a protein level was performed in F. N is 5 in each group. *P < .05 vs NC plus Vehicle. ^#^ *P* < .05 vs NC plus TGF‐β. G‐I, Cardiac fibroblasts were infected with adenovirus vector or expressing Prrx2 cDNA for 48 h. Cells were subjected to detect Wnt5a gene expression in G and protein level in H, and quantitative analysis of Wnt5a protein level was shown in I. N is 5 in each group. *P < .05 vs Vector

Considering that Prrx2 overexpression up‐regulates both the mRNA level and protein expression of Wnt5a, we have been suggested that Prrx2 may up‐regulate Wnt5a through transcriptional activation. By using the transcription factor database, we found that Wnt5a upstream promoter contains a putative Prrx2 binding site (ACAATTTC) as shown in Figure 6A. To confirm the predicted site in Wnt5a promoter is required for increased Wnt5a gene expression in cardiac fibroblasts in response to TGF‐β or Prrx2 overexpression, we constructed two promoter‐reporter plasmids containing different binding site of Wnt5a promoter (ACAATTTC and AGTTAAAC). HEK293 cells were transfected with wild‐type (WT) or mutated (MT) Wnt5a promoter‐reporter plasmids along with vector or Prrx2 cDNA. Promoter activity was analysed by luciferase assay in Figure 6B. Prrx2 overexpression increased the transcriptional activity of WT‐Wnt5a promoter. However, it had no any effects on the transcriptional activity of MT‐Wnt5a promoter, implying that the regulatory element necessary for Prrx2‐induced enhancement of Wnt5a gene expression is ACAATTTC.

The direct interaction between Prrx2 protein and Wnt5a promoter in cultured cardiac fibroblasts was determined by using immunoprecipitation (ChIP) analysis as described previously.^26^ Compared to vehicle‐treated cells, the Wnt5a gene promoter was positively amplified in samples from TGF‐β‐treated cells following ChIP with Prrx2 primary antibody but not with control IgG (Figure 6C), suggesting that the positive amplification of the Wnt5a gene promoter is specific to Prrx2.

### 3.9 TGF‐β via Prrx2 up‐regulates Wnt5a gene expression in cardiac fibroblasts

As seen in Figure 6D‐F, TGF‐β increased both mRNA and protein expressions of Wnt5a in cells infected with adenovirus expressing negative control shRNA. However, TGF‐β was not able to up‐regulate the expressional levels of Wnt5a mRNA and protein if cells expressed Prrx2 shRNA. Besides, we infected cardiac fibroblasts with adenovirus expressing Prrx2 cDNA for 48 hours and then detected Wnt5a gene expressions. Liking TGF‐β, Prrx2 overexpression up‐regulated Wnt5a mRNA and protein levels in cardiac fibroblasts (Figure 6G‐I), also seconding that Prrx2 is an upstream modulator of Wnt5a gene expression in cardiac fibroblasts.

### 3.10 TGF‐β via Prrx2‐Wnt5a signalling induces cell differentiation of cardiac fibroblasts in vitro

**Figure 7:**
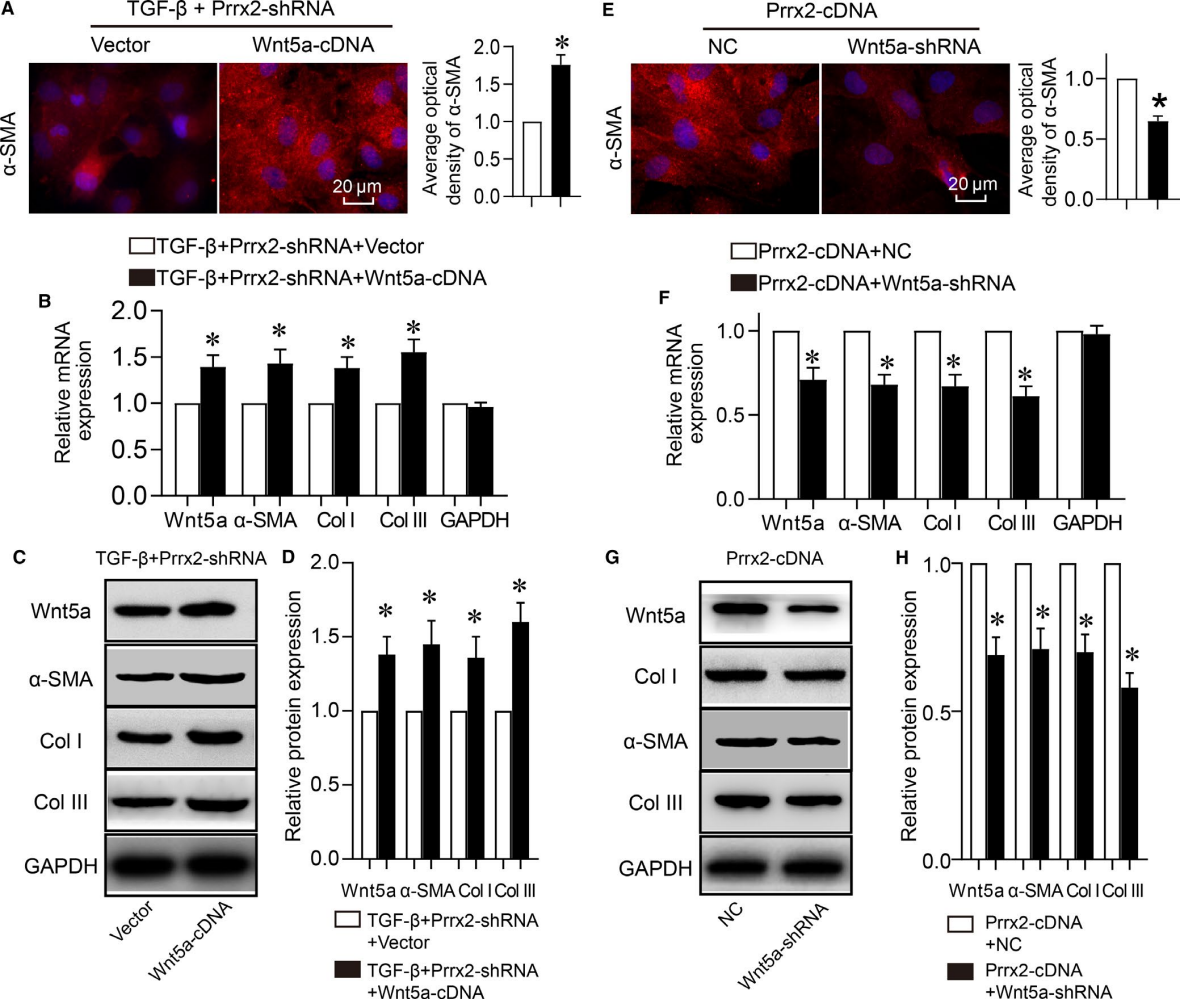
TGFβ1 via Prrx2‐Wnt5a signalling induces cell differentiation in cardiac fibroblasts. A‐D, Cultured cardiac fibroblasts were co‐infected with adenovirus expressing Prrx2 shRNA plus vector or Wnt5a cDNA for 48 h and then treated with TGF‐β (10 ng/mL) for 24 h. Cell differentiation was determined by immunofluorescence analysis of α‐SMA and quantitative analysis was shown in A. Gene expressions of Wnt5a, α‐SMA, GAPDH, collagen I (Col I) and collagen III (Col III) in cells were measured by real‐time PCR in B. Total cell lysates of cardiac fibroblasts were subjected to perform Western blotting analysis to detect protein levels of Wnt5a, α‐SMA, Col I, and Col III in C and quantitative analysis was performed in D. N is 5 in each group. *P < .05 vs Vector. E‐H, Cardiac fibroblasts were co‐infected with adenovirus expressing Prrx2 cDNA plus negative control (NC) shRNA or Wnt5a shRNA for 48 h. Cells were subjected to detect immunofluorescence analysis of α‐SMA and quantitative analysis was shown in E, gene expressions of Wnt5a, α‐SMA, GAPDH, Col I and Col III in F, protein levels of Wnt5a, α‐SMA, Col I, and Col III in G and H. N is 5 in each group. *P < .05 vs NC plus Prrx2

Knowing that TGF‐β via Prrx2 up‐regulates Wnt5a gene expression in cardiac fibroblasts, we finally tested if the Prrx2‐Wnt5a signalling mediates TGF‐β‐induced cell differentiation of cardiac fibroblasts. Though TGF‐β failed to induce cell differentiation if cardiac fibroblasts were infected with adenovirus expressing Prrx2 shRNA (Figure 6A‐D), overexpression of Wnt5a reversed TGF‐β‐induced these alterations, as determined by the elevated mRNA and protein levels of α‐SMA, collagen I and III in cardiac fibroblasts, if they were enforced to express exogenous Wnt5a (Figure 7A‐D). Further, cell differentiations induced by Prrx2 overexpression were rescued by adenovirus‐mediated gene knockdown of Wnt5a in cardiac fibroblasts (Figure 7E‐H).

## 4 DISCUSSION

In the present study, we provided the first evidence that TGF‐β up‐regulates Prrx2 to activate Wnt5a signalling through gene transcription, which induces cell differentiation of cardiac fibroblasts. As a result, this contributes to cardiac remodelling and the delayed recovery of heart functions after MI (Figure S4). This novel mechanism not only uncovers a molecular mechanism by which Wnt5a signalling is re‐activated in cardiac fibroblasts during ischaemia in vivo, but also provides a novel target for exploring new drugs to improve the prognosis of ischaemic heart disease.

The major discovery of this study is that Prrx2 regulates Wnt5a signalling via gene transcriptional manner in cardiac fibroblasts. To the best of our knowledge, this is the first study to identify Prrx2 as a transcriptional factor of Wnt5a, which was supported by the following evidence: (a) In Wnt5a gene promoter, the consensus sequence of Prrx2 is found, which is a putative Prrx2 binding site ‘ACAATTTC’; (b) Analysis of luciferase gene reporter told us that Prrx2 may interact with Wnt5a promoter and activate its transcriptional activity; (c) In cells treated with TGF‐β, ChIP analysis revealed the specific binding of Prrx2 with Wnt5a gene promoter if Prrx2 protein in nuclear lysates was purified by using primary Prrx2 antibody but not control IgG; (d) Importantly, both in vitro and in vivo gene knockdown of Prrx2 down‐regulated Wnt5a mRNA and protein levels, while Prrx2 overexpression enhanced Wnt5a gene expression in cultured cardiac fibroblasts. Although Wnt5a gene is regulated by several transcriptional factors, such as Egr‐1, Twist and NF‐κB,^27^, ^28^, ^29^ this is the first to ascertain that Prrx2 directly regulates Wnt5a gene transcription by binding to the promoter region of nucleotide sequence. Associated to that Wnt5a is involved in other biological functions, such as glucose homeostasis,^30^ embryonic development^31^ and multiple cancers,^32^ identification of Prrx2 as a novel Wnt5a transcriptional factor not only helps us to understand the molecular mechanism of cardiac remodelling, but also explores the novel role of Wnt5a in other aspects related to Prrx2.

Another discovery of this project is that MI‐induced cardiac fibrosis is Prrx2 dependent. Cardiac fibrosis is an important pathological process contributing to the pathogenesis of cardiac remodelling after MI, which is a transition from an early inflammatory phase to fibrotic granulation and maturation stage of cardiac remodelling.^6^ As a matter of fact, myocardial fibrosis is the end‐points of cell differentiation, activation and proliferation of cardiac fibroblasts.^33^, ^34^ Our study firstly explains the molecular mechanism by how TGF‐β induces cell differentiation of cardiac fibroblasts in myocardial remodelling through Prrx2 up‐regulation. As concerned to why Prrx2 gene expression is elevated in cardiac fibroblasts after MI, an underlying mechanism is through the production of TGF‐β, which is a key factor contributing to cardiac fibrosis in heart diseases^35^ and TGF‐β is able to induce both Prrx2 in cancer cells.^16^

An unanswered question in this study that how TGF‐β up‐regulates Prrx2 gene expression in cardiac fibroblasts. The gene function may be regulated in transcriptional, post‐transcriptional and post‐translational levels.^36^, ^37^ For example, microRNA (miR) comprises small, non‐coding RNA that binds to a specific 3’‐untranslated region of mRNA and inhibits mRNA translation or promotes mRNA degradation.^38^, ^39^ It has been reported that Prrx2 mRNA is a target of miR‐212‐5p in breast cancer.^40^ Interestingly, TGF‐β up‐regulates multiple microRNAs to produce its biological actions such as miR‐124 and miR‐101 as epigenetic factors.^41^, ^42^, ^43^ Thus, we reasoned that TGF‐β may increase Prrx2 expression via the specific miR. Further investigations are needed.

Interestingly, we observed that knockdown of Prrx2 did not affect basal gene expression of Wnt5a in cardiac fibroblasts. We reasoned these possibilities to produce this difference. First, another Prrx2‐induced transcription factor is promoting the increase instead of direct Prrx2 effect. Second, Prrx2 is partially involved in the cardiac fibrosis in resting condition.

An issue need to be discussed is why the death occurred at the first 10 days for MI mice. In the MI model alone, 32% of mice died of cardiac rupture and 12% mice died of heart failure. While in the MI model of mice with Prrx2 knockdown, 16% of mice died of cardiac rupture and 8% mice died of heart failure. Based on our investigations, we thought the major cause of death is cardiac rupture and Prrx2 knockdown can prevent cardiac rupture.

In summary, the present study proposes a role of Prrx2 in cardiac remodelling in response to ischaemic stress in heart. Specifically, when ischaemia is induced, Prrx2 is activated by TGF‐β to enhance Wnt5a signalling, which in turn induces cell differentiation. The inhibition of either Prrx2 or Wnt5a serves to maintain resting condition of cardiac fibroblasts, which is ultimately not enough for promoting cardiac fibrosis. In perspective, Prrx2 or Wnt5a inhibitor maybe a new drug to treat ischaemia‐related cardiovascular diseases, such as acute MI and stroke.

## CONFLICTS OF INTEREST

None.

## AUTHOR CONTRIBUTION

WWB performed all experiments and drafted the manuscript. ZYT, TCS, XJJ, PL, WDQ, PS and BW partially performed some experiments. JX, ZL, HYY and ZMM gave a lot of critical suggestions to this project. SXW, CL and TG conceived the idea, designed all experiments, convinced the whole project and revised the manuscript.

## Data Availability

The datasets used and analysed during the current study are available from the corresponding author on reasonable request.
