# Supplementary material for: Up‐regulation of paired‐related homeobox 2 promotes cardiac fibrosis in mice following myocardial infarction by targeting of Wnt5a
Source: J Cell Mol Med. 2019 Dec 27;24(3):2319–29. doi: 10.1111/jcmm.14914 (PMC7011146; doi:10.1111/jcmm.14914)
Supplement: Supplementary file 1 [file JCMM-24-2319-s001.doc]

**Supplementary Materials for**

**Upregulation of paired-related homeobox 2 promotes cardiac fibrosis in mice following myocardial infarction by targeting of Wnt5a**

Wen-Wu Bai^1,2^, Zhen-Yu Tang^3^, Ti-Chao Shan^4^, Xue-Jiao Jing^5^, Peng Li^6^, Wei-Dong Qin^4^, Ping Song^6^, Bo Wang^2^, Jian Xu^6^, Zhan Liu^7^, Hai-Ya Yu^8^, Zhi-Min Ma^9^, Shuang-Xi Wang^1,6,*^, Chao Liu^8,10,*^, Tao Guo^1,*^

^1^The Key Laboratory of Cardiovascular Remodeling and Function Research, Chinese Ministry of Education, Chinese National Health Commission and Chinese Academy of Medical Sciences, The State and Shandong Province Joint Key Laboratory of Translational Cardiovascular Medicine, ^2^Department of Traditional Chinese Medicine, ^3^Department of Emergency, ^4^Department of Critical Care Medicine, and ^5^Department of Healthcare, Qilu Hospital of Shandong University, Jinan, China; ^6^Department of Pharmacology, College of Pharmacy, Xinxiang Medical University, Xinxiang, China; ^7^Department of Gastroenterology and Clinical Nutrition, The First Affiliated Hospital of Hunan Normal University, Changsha, China; ^8^Department of Neurology, The People’s Hospital of Xishui County, Huangang, Hubei, China; ^9^Department of Endocrinology, The Affiliated Suzhou Science &Technology Town Hospital of Nanjing Medical University, Suzhou, China; ^10^Hubei Key Laboratory of Cardiovascular, Cerebrovascular, and Metabolic Disorders, Hubei University of Science and Technology, Xianning, China

^*^Correspondence should be addressed to Shuang-Xi Wang, Chao Liu, and Tao Guo, 107 Wenhua Xi Road, Jinan, 250012 China. Tel. 86-531-82169240. Fax 86-531-82169259. Email shuangxiwang@sdu.edu.cn, xn_liuchao@163.com, and guotao@sdu.edu.cn

**Supplementary Methods and Materials**

**Reagents**

Polyclonal or monoclonal antibodies against Wnt5a, p-ERK, p-JNK, GAPDH, and α-SMA were obtained from Cell Signaling Company. Primary Prrx2 antibody was purchased from Sigma Company. Collagen I and collagen III antibodies were from Abacm. Recombinant wnt5a protein was purchased from Stem RD (Cat# W5A-M-005). All drug concentrations were expressed as the final molar concentration in the buffer.

**Animals and experimental protocols**

Male *Apoe^-/-^* mice,6-8 weeks of age, were obtained from Beijing Huafukang Animal Experimental Center. Mice were housed in temperature-controlled cages with a 12-h light-dark cycle and given free access to water and a high-fat diet (0.25% cholesterol and 15% cocoa butter) for 8 weeks. This animal study was carried out instrict accordance with the recommendations in the Guide for the Care and Use of Laboratory Animals of the National Institutes of Health. The animal protocol was reviewed and approved by the University of Shandong, Animal Care and Use Committee.

Our study in animal models consisted of two parts. In the first part of the animal study, mice received MI surgery and raised for 30 postoperative days. At the 30^th^ postoperative day, echocardiography was performed to assess heart functions. At the end of experiment, all mice were sacrificed under anesthesia by intraperitoneal injection of 0.8% pentobarbital sodium (60 mg/kg).

In the second part of the animal study, mice received tail vein injection of adenovirus expressing negative control or Prrx2 shRNA. For infection, virus was injected in 100 μl of PBS containing 7.6X10^7^ PFU. Three days after virus infection, echocardiography was performed to assess heart function followed by MI surgery. At the 30^th^ postoperative day, echocardiography was performed again to assess heart function. At the end of experiment, all mice were sacrificed under anesthesia by intraperitoneal injection of 0.8% pentobarbital sodium (60 mg/kg).

**Myocardial infarction**

1. Sterilize surgical instruments with a dry bead sterilizer (Germinator 500).

2. All mice (aged 8-12 weeks) were anesthetized with 2-3% isoflurane inhalation in an inducing chamber.

3. Once anesthetized, the mouse is removed from the inducing chamber to the surgical board, immobilized with tape, and continuously anesthetized with 2% isoflurane via coaxial breathing apparatus but not ventilated.

4. Remove the fur with a standard depilatory (e.g., Nair) and clean the skin with water and then betadine and alcohol pads. In order to perform this procedure more efficiently, the step of fur-removing could be done earlier.

5. Two small incisions (0.5 cm long) are made on the left and right chest skin with the scissors to expose the 3rd intercostal space.

6. Echocardiography is performed using a VEVO 2100 imaging system (Visual Sonics Inc., Toronto, Canada) with a 30 MHz phased array transducer and a frame rate of 235/s. The echocardiography probe (MS-400) is placed perpendicular to the sagittal plane of the chest within the 3rd intercostal space, imaging the left ventricle (LV) short axis.

7. A small straight needle (0.2 mm in diameter) was inserted at the costal angle of the superior margin of the 3^rd^ rib in the left chest. Under the guide of ultrasound, the heart is punctured in the inferior of left anterior descending coronary artery (LADCA) by an 8-0 silk suture attached to the needle. The needle is coming out of skin from the right chest.

8. Then, the needle is inserted back from the right to of the left. When the needle passes through the heart, it goes through above LADCA under ultrasound and came out the skin from the same site in the left chest.

9. Once a loose knot is made, the needle is inserted back from the left to the right in the chest. The LADCA is now located inside of the knot.

10. Ligation of LADCA by pulling the two ends of the suture carefully. The ischemia was confirmed by the elevation of ST segment recorded by the echocardiography imaging system during the surgery. The knot is readily visible under ultrasound.

11. The mouse is then allowed to breathe room air and monitored on a heating blanket during the recovery period, which is generally complete within 3-5 min.

12. The sham group undergoes the same surgical procedures except that the LADCA is not occluded.

13. One dose of buprenorphine (0.1 mg/kg) is administered subcutaneously (s.c.) immediately after the incision is closed.

**Echocardiography**

Echocardiography was performed as described previously. Echocardiography was performed with a Vevo2100 Imaging System (Visual Sonics Inc.) with a MS-400 ultrasound transducer. After anesthetization (2% inhaled isoflurane), the mouse’s left ventricular (LV) was assessed in both parasternal short-axis and long-axis view. The end-systole and end-diastole were defined as the phase in which the smallest or largest LV area was obtained, respectively. All echocardiography was performed by the same investigator who was blinded to the experimental groups. EF, Ejection fractions. FS, Fractional shortening. LVDd, Diastolic left ventricular internal diameter. LVDs, Systolic left ventricular internal diameter.

**Masson’s Trichrome Staining**

As described previously, heart samples were fixed in 4% PFA and then embedded in paraffin. Five μm-thick sections were subjected to Masson’s trichrome staining following a standard procedure. Images of the left ventricular area of each section were taken (200X magnification) with Spot Insight camera. Image J Software (National Institutes of Health) was used to quantify fibrotic region in each section. The percentage of fibrosis was measured as fibrosis areas/total left ventricular areas X 100%.

**Generation of shRNA construct and adenovirus production**

Based on the protocol from Signaling Gateway, the shRNA cassette containing target sequence of Prrx2/Wnt5a was designed. The cassette was subcloned into pEN-hH1c vector as described previously. And then the pEN-hH1c vector containing the Prrx2/Wnt5a shRNA cassette was combined with an attR-containing vector pDSL-hpUP in an LR recombination reaction. The recombinant constructs pDSL-hpUP-Prrx2/Wnt5a-shRNA was confirmed by DNA sequence analysis. The sequence of Prrx2 shRNA is CCGGCCGGAGGTTCAAGTCCTGCAACTCGAGTTGCAGGACTTGAACCTCCGGTTTTTG. The sequence of Wnt5a shRNA is CCGGGCTAATTCTTGGTGGTCTCTACTCGAGTAGAGACCACCAAGAATTAGCTTTTTG. The sequence of negative control shRNA is TTCTCCGAACGTGTCACGT. The adenovirus was produced by transiently transfecting HEK293T cells using Super Fect transfection reagent (Qiagen, USA) with three packing plasmid system (pGag/Pol,pRev,andpVSV-G). The virus-containing supernatant was collected 72 hours after transfection, and filtered through 0.45 mm filters (Millipore, USA), and stored at -80°C. The titer of the viral vectors was determined by TCID50 (Tissue culture infective dose) method.

**Generation of cDNA construct and adenoviral infection to cells**

To generate adenoviral vector containing Prrx2/Wnt5a cDNA, we subcloned a murine cDNA encoding full-length of Prrx2/Wnt5a into a shuttle vector (pShuttle CMV [cytomegalovirus]) as described previously. Cells were infected with adenovirus overnight in medium supplemented with 2% FBS. The cells were then washed and incubated in fresh medium for an additional 48 hours before experimentation. These conditions typically produced an infection efficiency of at least 80%.

Isolation and culture of cardiac fibroblasts

Fibroblasts were isolated from mice hearts as previously described. Cardiac fibroblasts were cultured in Dulbecco’s modified Eagle’s medium (DMEM, HyClone) supplemented with 10% fetal bovine serum in a humidified atmosphere with 5%CO_2_ at 37°C.Only cells with no more than three passages were used in this study.

**RNA quantifications by RT-qPCR**

Total RNA was isolated using a TRIzol-based (Invitrogen)RNA isolation protocol. For mRNA detections, the iScript cDNA Synthesis Kit (Bio-Rad) was used to synthesize first-strand cDNA according to the manufacturer’s protocol. Reactions were run for 30 cycles at conditions as follows: denaturation for 30 seconds at 94°C, annealing for 30 seconds at 57°C, and extension for 30 seconds at 72°C. Constitutively expressed GADPH mRNA was amplified as control. All primers were presented in Suppl. Table S1.

**Western blot**

Cells or tissues were homogenized on ice in cell-lysis buffer containing 20 mM Tris-HCl (pH 7.5), 150 mM NaCl, 1 mM Na_2_EDTA, 1 mM EGTA, 1% Triton, 2.5 mM sodium pyrophosphate, 1 mM beta-glycerophosphate, 1 mM Na_3_VO_4_, 1 µg/ml leupeptin, and 1 mM PMSF. Protein samples were solubilized in SDS sample buffer, and 20 µg of protein was separated by SDS-PAGE using 8-10% polyacrylamide gels, transferred to nitrocellulose membranes. Entire sheets of hybond-ECL membranes containing transferred proteins were incubated firstly in 5% non-fat dry milk for 2 hours to block nonspecific binding of antibodies, followed by overnight incubation in primary antibodies diluted 1:1000 at 4°C. The membranes were then washed 3 times with TBST and incubated for 1 hour with second antibody diluted 1:5000 at room temperature. Bound antibodies were detected with ECL-enhanced chemiluminescence (Amersham Biosciences) according to the manufacturer's protocols and analyzed by the use of Image-Pro Plus 6.0. Background intensity was subtracted from all calculated areas and we used the ratio of control group as 1 as described previously.

**Generation of Wnt5a gene promoter mutant and plasmid transfection into HEK293, and reporter assays**

“ACAATTTC” in wildtype Wnt5a gene promoter (WT-Wnt5a) was mutated to “AGTTAAAC” (MT-Wnt5a) by using the QuikChange kit (Stratagene), according to the manufacturer's instructions. Plasmid DNAs (WT-Wnt5a and MT-Wnt5a) were subcloned the pCMV β-gal plasmid and co-transfected into HEK293 cells with Prrx2 cDNA by using the Lipofectamine 2000 kit (Invitrogen, catalog no. 11668-019). Cells were harvested 48 hours after transfection, and luciferase and β-galactosidase activities were measured.

**Immunofluorescence (IFC)**

After treatment, cells on sterile glass cover slips were rinsed by cold PBS and then fixed by incubation with 10% formalin in PBS for 10 minutes. Block cells by 5% BSA for 30 minutes. Incubate cells with primary antibody for 1 hour at room temperature or overnight at 4^o^C. After washing, incubate with fluorescence-conjugated secondary antibody for 45 minutes. Digital images were captured under a fluorescence microscopy. Quantitative analysis of a-SMA by calculating fluorescence intensity using Alpha Ease FC software (version 4.0 Alpha Innotech). The control group was set up as 1.

**ChIP assay for Prrx2 and Wnt5a gene promoter binding**

ChIP assays were performed according to the manufacturer's protocol by using a ChIP kit. 1X10^6^ cells were seeded on a 10 cm dish. Histones were cross-linked to DNA by adding formaldehyde directly to culture medium at a final concentration of 1% and incubating for 10 min at 37ºC. The cells were harvested in SDS lysis buffer and added protease inhibitors. Cell lysates were sonicated to shear DNA to lengths between 200 and 1000 bp. Sheared chromatin was precleared with protein G beads prior to incubation overnight at 4 °C with 4 µg of anti-Prrx2 antibody or control IgG. Purified, immunoprecipitated chromatin fragments from IP samples were subjected to PCR. PCR amplification of the Wnt5a gene promoter was performed with primers of the Wnt5a promoter. PCR products were subjected to 2% agarose gel electrophoresis and stained with ethidium bromide.

**Biochemical studies**

Fasting blood samples of mice were collected before euthanasia. Serum concentrations of total cholesterol (TC), triglycerides (TG), low-density lipoprotein cholesterol (LDL), and high-density lipoprotein cholesterol (HDL) were determined by a commercially available enzymatic assay using a biochemistry automatic analyzer (HITACHI 7170A, Hitachi, Tokyo, Japan).

**Statistical analysis**

All quantitative results are expressed as mean ± SD. The normal distribution of data was tested by the Kolmogorov-Smirnov test before statistical comparisons, and the normality/equal variance was tested to determine whether ANOVA was appropriate. Multiple comparisons were analyzed with a one-way ANOVA followed by Tukey *post-hoc* tests or Bonferroni *post-hoc* analyses. Comparisons between two groups were analyzed by unpaired Student's *t* test between two groups. Chi-Square test was applied to comparisons of survival rates. Statistical analyses were conducted using GraphPad Prism 6.0 or IBM SPSS statistics 20.0. A two-sided *P*-value<0.05 was considered significant.

**References**

1. **Yang J, Liu X, Jiang G, Chen Y, Zhang Y, Zhang M.** Two-dimensional strain technique to detect the function of coronary collateral circulation. *Coron Artery Dis*. 2012; 23: 188-94.

2. **Bai WW, Xing YF, Wang B, Lu XT, Wang YB, Sun YY, Liu XQ, Guo T, Zhao YX.** Tongxinluo improves cardiac function and ameliorates ventricular remodeling in mice model of myocardial infarction through enhancing angiogenesis. *Evidence-based complementary and alternative medicine : eCAM*. 2013; 2013: 813247.

3. **Godec J, Cowley GS, Barnitz RA, Root DE, Sharpe AH, Haining WN.** Inducible RNAi in vivo reveals that the transcription factor BATF is required to initiate but not maintain CD8+ T-cell effector differentiation. *Proc Natl Acad Sci U S A*. 2015; 112: 512-7.

4. **Wang S, Zhang M, Liang B, Xu J, Xie Z, Liu C, Viollet B, Yan D, Zou MH.** AMPKalpha2 deletion causes aberrant expression and activation of NAD(P)H oxidase and consequent endothelial dysfunction in vivo: role of 26S proteasomes. *Circ Res*. 2010; 106: 1117-28.

5. **Garate-Carrillo A, Ramirez I.** Embryonary Mouse Cardiac Fibroblast Isolation. *Methods Mol Biol*. 2018; 1752: 71-9.

6. **Wang S, Xu J, Song P, Wu Y, Zhang J, Chul Choi H, Zou MH.** Acute inhibition of guanosine triphosphate cyclohydrolase 1 uncouples endothelial nitric oxide synthase and elevates blood pressure. *Hypertension*. 2008; 52: 484-90.

7. **Wang S, Xu J, Song P, Viollet B, Zou MH.** In vivo activation of AMP-activated protein kinase attenuates diabetes-enhanced degradation of GTP cyclohydrolase I. *Diabetes*. 2009; 58: 1893-901.

8. **Xie Z, Dong Y, Zhang J, Scholz R, Neumann D, Zou MH.** Identification of the serine 307 of LKB1 as a novel phosphorylation site essential for its nucleocytoplasmic transport and endothelial cell angiogenesis. *Mol Cell Biol*. 2009; 29: 3582-96.

9. **Park MJ, Kwak HJ, Lee HC, Yoo DH, Park IC, Kim MS, Lee SH, Rhee CH, Hong SI.** Nerve growth factor induces endothelial cell invasion and cord formation by promoting matrix metalloproteinase-2 expression through the phosphatidylinositol 3-kinase/Akt signaling pathway and AP-2 transcription factor. *J Biol Chem*. 2007; 282: 30485-96.


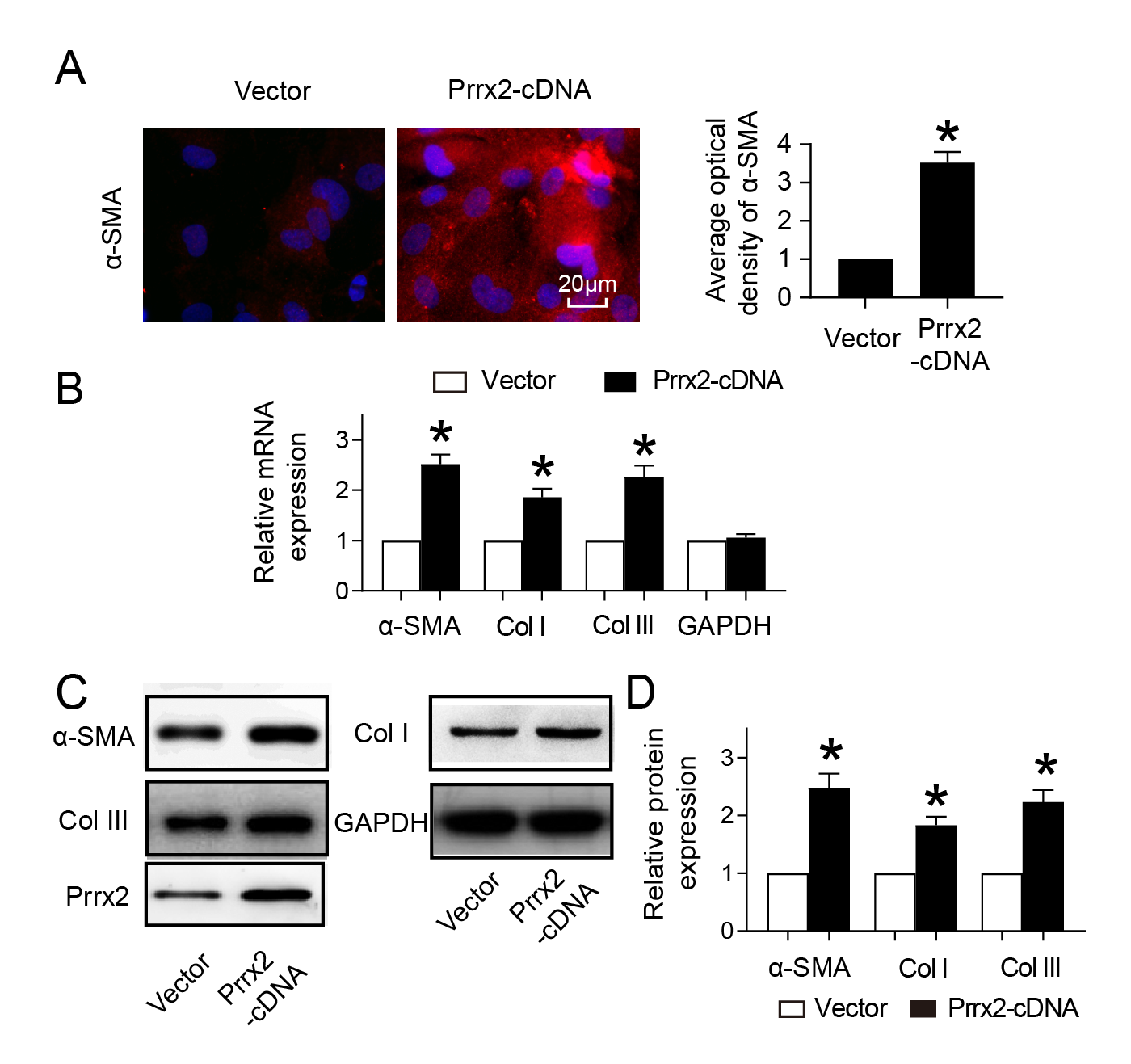


**Supplementary Figure S1. Overexpression of Prrx2 induces cell differentiation in cardiac fibroblasts.** Cultured cardiac fibroblasts were infected with adenovirus expressing vector or Prrx2 cDNA for 48 hours. (**A**) Cell differentiation was determined by immunofluorescence analysis of α-SMA and quantitative analysis was shown. (**B**) Gene expressions of α-SMA, GAPDH, collagen I (Col I), and collagen III (Col III) in cells were measured by real-time PCR. (**C** and **D**) Total cell lysates of cardiac fibroblasts were subjected to perform Western blotting analysis to detect protein levels of α-SMA, Col I, and Col III in **C** and quantitative analysis was performed in **D**. N is 5 in each group. ^*^*P*<0.05 vs. Vector.


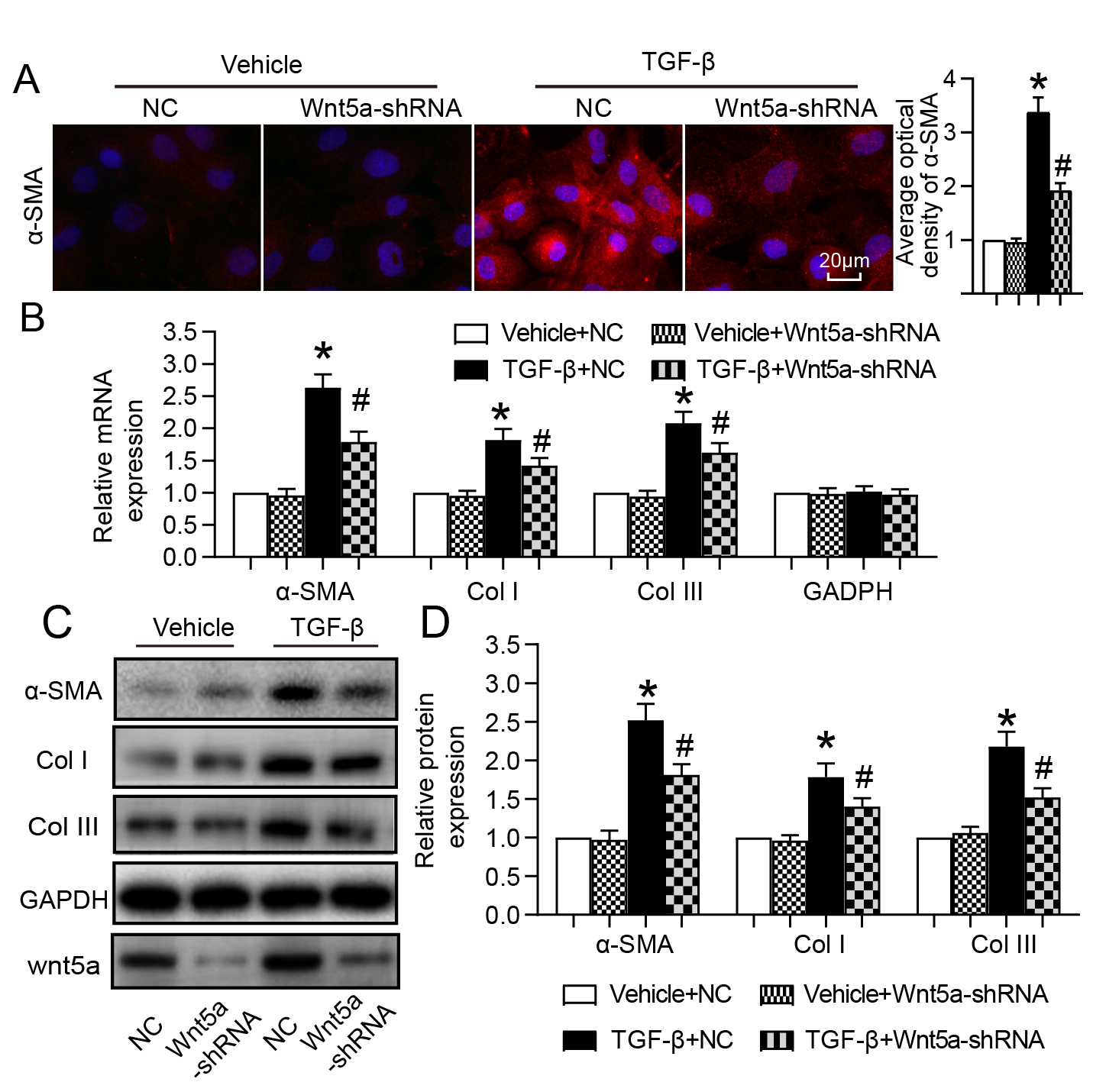


**Suppl. Figure S2. TGF-β induces cell differentiation via Wnt5a signaling in cardiac fibroblasts**. Cultured cardiac fibroblasts were infected with adenovirus expressing negative control (NC) shRNA or Wnt5a shRNA for 48 hours and thentreated with TGF-β (10 ng/ml) for 24 hours. (**A**) Cell differentiation was determined by immunofluorescence analysis of α-SMA and quantitative analysis was shown. (**B**) Gene expressions of α-SMA, GAPDH, collagen I (Col I), and collagen III (Col III) in cells were measured by real-time PCR. (**C** and **D**) Total cell lysates of cardiac fibroblasts were subjected to perform Western blotting analysis to detect protein levels of α-SMA, Col I, and Col III in **C** and quantitative analysis was performed in **D**. N is 5 in each group. ^*^*P*<0.05 vs. NC plus Vehicle. ^#^*P*<0.05 vs. NC plus TGF-β.

**
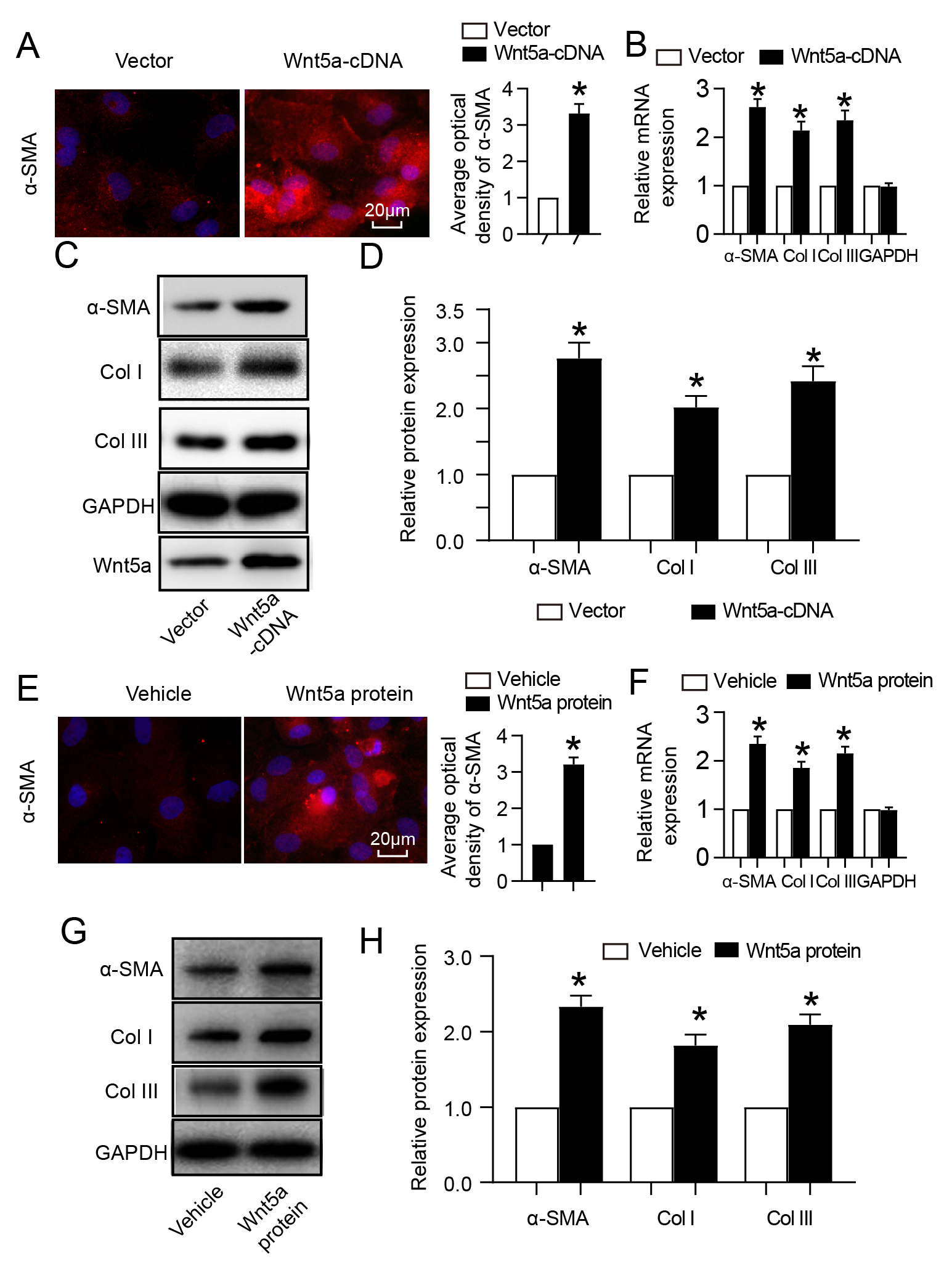
**

**Supplementary Figure S3. Overexpression of Wnt5a induces cell differentiation in cardiac fibroblasts**. (**A-D**) Cultured cardiac fibroblasts were infected with adenovirus expressing vector or Wnt5acDNAfor48hours. (**A**) Cell differentiation was determined by immunofluorescence analysis of α-SMA and quantitative analysis was shown. (**B**) Gene expressions of α-SMA, GAPDH, collagen I (Col I), and collagen III (Col III) in cells were measured by real-time PCR. (**C** and **D**) Total cell lysates of cardiac fibroblasts were subjected to perform Western blotting analysis to detect protein levels of α-SMA, Col I, and Col III in **C** and quantitative analysis was performed in **D**. N is 5 in each group. ^*^*P*<0.05 vs. Vector. (**E-H**) Cultured cardiac fibroblasts were treated with recombinant wnt5a at 100 ng/ml for 24 hours. (**E**) Cell differentiation was determined by immunofluorescence analysis of α-SMA and quantitative analysis was shown. (**F**) Gene expressions of α-SMA, GAPDH, collagen I (Col I), and collagen III (Col III) in cells were measured by real-time PCR. (**G** and **H**) Total cell lysates of cardiac fibroblasts were subjected to perform Western blotting analysis to detect protein levels of α-SMA, Col I, and Col III in **C** and quantitative analysis was performed in **D**. N is 5 in each group. ^*^*P*<0.05 vs. Vehicle.

**
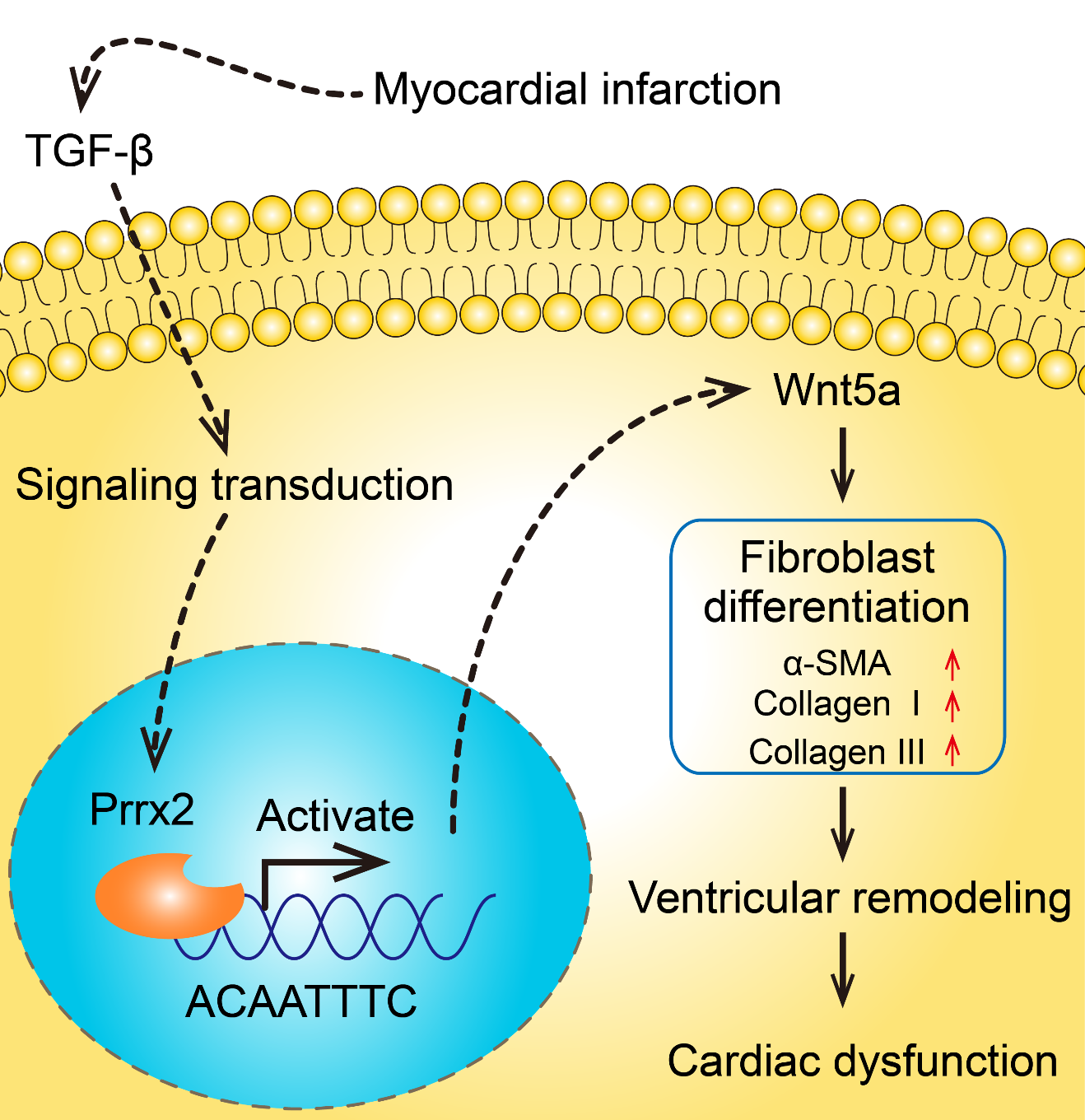
**

**Supplementary Figure S4. Proposed mechanism of cardiac fibrosis after ischemia.** In the post-ischemic heart, TGF-β activates Prrx2 to upregulate Wnt5a gene transcription through interacting with Wnt5a promoter. Activated Wnt5a signaling induces cell differentiation of cardiac fibroblasts, contributing to cardiac remodeling and the delayed recovery of heart function.

**Supplementary Table S1.** Primers used in this project.

| CHIP Assay Primer Sequences | | | |
| --- | --- | --- | --- |
|  | | Forward Primer Sequence (5' - 3') | Reverse Primer Sequence (5' - 3') |
| **Wnt5a**  **promoter** | | TAGGGTGGTGCTTATCCATCC | GAAGTTTAGGTGAACCGGGGA |
| RT-RCR Primer Sequences | | | |
| Gene | Forward Primer Sequence (5' - 3') | | Reverse Primer Sequence (5' - 3') |
| Prrx2 | CGTGGCACCAAACGAAAGAAG | | GTAGTGTGTGCGCTCAAATACA |
| Wnt5a | CAACTGGCAGGACTTTCTCAA | | CATCTCCGATGCCGGAACT |
| α-SMA | GTCCCAGACATCAGGGAGTAA | | TCGGATACTTCAGCGTCAGGA |
| Col I | GCTCCTCTTAGGGGCCACT | | CCACGTCTCACCATTGGGG |
| Col III | CTGTAACATGGAAACTGGGGAAA | | CCATAGCTGAACTGAAAACCACC |
| TGF-β1 | CTCCCGTGGCTTCTAGTGC | | GCCTTAGTTTGGACAGGATCTG |
| GAPDH | AGGTCGGTGTGAACGGATTTG | | TGTAGACCATGTAGTTGAGGTCA |

**Supplementary Table S2. Characteristics of mice *in vivo*** study: part one

| Parameters | Sham | MI |
| --- | --- | --- |
| Weight ( g ) | 28.72 ± 1.04 | 27.35 ± 0.81 |
| TC ( mmol/l ) | 26.32 ± 2.16 | 27.19 ± 1.78 |
| TG ( mmol/l ) | 0.87 ± 0.08 | 0.94 ± 0.11 |
| HDL-C ( mmol/l ) | 4.65 ± 0.36 | 4.74 ± 0.45 |
| LDL-C ( mmol/l ) | 3.33 ± 0.31 | 3.42 ± 0.26 |

**Note:** total cholesterol (TC), triglycerides (TG), low-density lipoprotein cholesterol (LDL), and high-density lipoprotein cholesterol (HDL) were determined by a commercially available enzymatic assay using a biochemistry automatic analyzer (HITACHI 7170A, Hitachi, Tokyo, Japan).

**Supplementary Table S3. Characteristics of mice *in vivo*** study: part two

| Parameters | Sham+NC | Sham+Prrx2-shRNA | MI+NC | MI+Prrx2-shRNA |
| --- | --- | --- | --- | --- |
| Weight( g ) | 28.56 ± 1.25 | 27.83 ± 1.32 | 27.47 ± 0.92 | 28.25 ± 0.86 |
| TC( mmol/l ) | 25.87 ± 2.03 | 26.47 ± 2.36 | 27.13 ± 1.82 | 26.19 ± 1.67 |
| TG( mmol/l ) | 0.85 ± 0.09 | 0.92 ± 0.08 | 0.91 ± 0.12 | 0.86 ± 0.10 |
| HDL-C( mmol/l ) | 4.85 ± 0.26 | 4.96 ± 0.35 | 5.22 ± 0.43 | 5.15 ± 0.47 |
| LDL-C( mmol/l ) | 3.43 ± 0.22 | 3.53 ± 0.25 | 3.62 ± 0.27 | 3.58 ± 0.26 |

**Note:** total cholesterol (TC), triglycerides (TG), low-density lipoprotein cholesterol (LDL), and high-density lipoprotein cholesterol (HDL) were determined by a commercially available enzymatic assay using a biochemistry automatic analyzer (HITACHI 7170A, Hitachi, Tokyo, Japan).
